# Large Language Models and the Analyses of Adherence to Reporting Guidelines in Systematic Reviews and Overviews of Reviews (PRISMA 2020 and PRIOR)

**DOI:** 10.1007/s10916-025-02212-0

**Published:** 2025-06-12

**Authors:** Diego A. Forero, Sandra E. Abreu, Blanca E. Tovar, Marilyn H. Oermann

**Affiliations:** 1 School of Health and Sport Sciences, Fundación Universitaria del Área Andina, Bogotá, Colombia; 2 Psychology Program, Fundación Universitaria del Área Andina, Medellín, Colombia; 3 Nursing Program, School of Health and Sport Sciences, Fundación Universitaria del Área Andina, Bogotá, Colombia; 4 Duke University School of Nursing, Durham, NC USA

**Keywords:** Reporting guidelines, Systematic reviews, Overview of reviews, Umbrella reviews, Evidence-based practice, Generative artificial intelligence, Meta-research

## Abstract

In the context of Evidence-Based Practice (EBP), Systematic Reviews (SRs), Meta-Analyses (MAs) and overview of reviews have become cornerstones for the synthesis of research findings. The Preferred Reporting Items for Systematic Reviews and Meta-Analyses (PRISMA) 2020 and Preferred Reporting Items for Overviews of Reviews (PRIOR) statements have become major reporting guidelines for SRs/MAs and for overviews of reviews, respectively. In recent years, advances in Generative Artificial Intelligence (genAI) have been proposed as a potential major paradigm shift in scientific research. The main aim of this research was to examine the performance of four LLMs for the analysis of adherence to PRISMA 2020 and PRIOR, in a sample of 20 SRs and 20 overviews of reviews. We tested the free versions of four commonly used LLMs: ChatGPT (GPT-4o), DeepSeek (V3), Gemini (2.0 Flash) and Qwen (2.5 Max). Adherence to PRISMA 2020 and PRIOR was compared with scores defined previously by human experts, using several statistical tests. In our results, all the four LLMs showed a low performance for the analysis of adherence to PRISMA 2020, overestimating the percentage of adherence (from 23 to 30%). For PRIOR, the LLMs presented lower differences in the estimation of adherence (from 6 to 14%) and ChatGPT showed a performance similar to human experts. This is the first report of the performance of four commonly used LLMs for the analysis of adherence to PRISMA 2020 and PRIOR. Future studies of adherence to other reporting guidelines will be helpful in health sciences research.

**Supplementary Information:**

The online version contains supplementary material available at 10.1007/s10916-025-02212-0.

## Clinical Trial Number

Not applicable.

**Supplementary Information:**

The online version contains supplementary material available at 10.1007/s10916-025-02212-0.

## Introduction

Evidence-Based Practice (EBP) has changed many aspects of the practice, research and teaching in medicine and other health sciences [1]. In this context, Systematic Reviews (SRs) and Meta-Analyses (MAs) have become cornerstones for the synthesis of research findings (for different types of primary studies) [2–4], being among the most highly cited articles [5] and are major inputs for clinical guidelines [6].

More recently, overviews of reviews, also called *umbrella reviews* [7], have emerged as novel types of articles, summarizing the results from multiple SRs and MAs and being useful for the research synthesis of entire topics and fields [8]. Reporting guidelines have been developed for multiple types of studies in the health sciences to ensure complete and transparent reporting [9] and in recent years, the Preferred Reporting Items for Systematic Reviews and Meta-Analyses (PRISMA) 2020 statement [10] and the Preferred Reporting Items for Overviews of Reviews (PRIOR) statement [11] are recommended for reporting SRs/MAs and overviews of reviews, respectively. Although these statements are robust, they require careful application and critical evaluation, to adapt to new challenges and advances in the health sciences.

In recent years, advances in Generative Artificial Intelligence (genAI) have been proposed as a potential major paradigm shift in scientific research [12]. In this context, Large Language Models (LLMs) have been explored for use in several processes employed in epidemiological research [13, 14], highlighting the need for their analysis of adherence to major reporting guidelines in health sciences research.

The automated analysis of adherence of reporting guidelines will be useful for meta-research works [15], as it will decrease the time needed to carry out these labor and time-intensive activities (given the large number of items to be extracted), allowing for much larger sizes of analyzed studies [13]. Those large studies of adherence might be a major input for efforts aimed at creating or modifying reporting guidelines [9], particularly for other type of studies (such as those for -omics sciences). In addition, the automated analysis of adherence will be helpful for authors of SRs/MAs and overviews of reviews to easily analyze, or improve, the adherence in their manuscripts before submission [16].

The main aim of this work was to examine the performance of four LLMs for the analysis of adherence to PRISMA 2020 and PRIOR, in a sample of SRs and overviews of reviews.

## Methods

In the current study, we tested the free versions of four commonly used LLMs: ChatGPT (GPT-4o) [17], DeepSeek (V3) [18], Gemini (2.0 Flash) [19] and Qwen (2.5 Max) [20]. Further details of the LLMs are described in Table S1. These four LLMs were selected as they are broadly used, free and allow the uploading of pdf files. As the chatbots for these LLMs were used, there was no option to modify parameters, such as those minimizing randomness.

An initial pilot phase was carried out to create working prompts for the LLMs and for both PRISMA 2020 and PRIOR. It involved several phases of refinement [21, 22], testing the improved versions of the prompts for each of the LLMs, and using three articles for both reporting guidelines. The selected prompts were chosen after complete answers were provided by the LLMs, simulating a real-word deployment [23] by health sciences researchers. The authors of this study include PhD-level experts in several fields of health sciences research.

The choice of the PRISMA 2020 and PRIOR statements is based on their recognition as highly used reporting guidelines of SRs/MAs and overviews of reviews, respectively. The exploration of the analysis of adherence by LLMs is of potential interest for many researchers around the world, as it would decrease the time needed to carry out these labor and time-intensive activities (given the large number of items to be extracted), allowing for much larger sizes of analyzed studies [24].

The PRISMA 2020 statement was published in 2021 [10] and is a widely used guideline for the reporting of SRs and MAs. PRISMA 2020 contains 27 items (with a total of 42 subitems; compliance with a larger number of items means a higher adherence), for the different sections of the SRs, such as Title, Abstract, Introduction, Methods, Results and Discussion). It also includes a commonly used flow diagram, which indicates the number of identified and included primary studies in the SRs [10].

The PRIOR statement was published in 2022 [11] and provides a guideline for reporting overviews of reviews, particularly related to health interventions. PRIOR contains 27 items (with a total of 46 subitems; compliance with a larger number of items means a higher adherence) and, similar to PRISMA 2020, it involves multiple aspects related to the different sections of the overviews of reviews and a flow diagram [11].

In order to have definitions of adherence to PRISMA 2020 and PRIOR, carried out by human experts, published studies about these were searched and identified. The supplementary files of Qin et al. [25] (for PRISMA 2020) and Lu et al. [26] (for PRIOR) were used to select randomly 20 SRs/MAs [27] and 20 overviews of reviews (Lists of the included studies are available in Supplementary file 1; the sample size of 20 has been previously used in other similar articles [22, 27, 28]). Qin et al. [25] and Lu et al. [26] were focused on SRs and overviews of reviews in the field of acupuncture and the adherence to PRISMA 2020 and PRIOR were carried out by the consensus of several experts in health sciences research. These two datasets were selected as they provided the complete information for each one of the included articles. The *RAND* function of the MS Excel 365 software (Microsoft Corporation, Redmond, WA) was used for the generation of random numbers, to select the studies to be included. The pdf files with the full text for each one of the selected SRs and overviews of reviews were uploaded (in March 2025) to each one of the LLMs, using the optimized prompts (Supplementary file 2), and their responses (including the explanations for their assessments) were retrieved and stored.

For the statistical analysis, several complementary approaches were used [21]. Based on previous studies [29, 30], for the determination of overall adherence for both PRISMA 2020 and PRIOR, each item was defined by each LLM as having adherence (reported), no adherence (not reported) or partial adherence (partially reported) and were counted as 1, 0 or 0.5, respectively, as previously done [26]. The overall adherence percentage was defined as the total sum divided by the total number of subitems (42 for PRISMA 2020; 46 for PRIOR), multiplied by 100.

The Shapiro–Wilk test was used to explore the normality of the studied numerical variables [31]. ANOVA tests [32] were carried out, followed by Tukey´s tests for adjustment [33], to determine statistical differences in the adherence scores determined by the LLMs and the human experts. A Pearson´s r coefficient [34] was calculated to determine the correlation between the responses of the LLMs and the human experts. The Altman-Bland plots and tables [35] were calculated to analyze in detail the agreement between each of the LLMs and the human experts (the mean differences and the confidence interval: +/- 1.96 SDs of the differences). Additionally, as previously described [36], the accuracy for each of the LLMs was also estimated. A *p* value < 0.05 was defined as statistically significant.

In addition, the calculation of adherence percentages to sections of PRISMA 2020 and PRIOR (such as for Methods and Results, among others) were also carried out and some examples of texts were extracted to visualize details of the responses generated by the LLMs. We did not calculate parameters such as specificity and sensitivity as they are used for topics with dichotomic variables [37]. The JASP program (version 0.18.3.0) [38] was employed for the statistical analyses. For the reporting of this study, we took into consideration key aspects of the MI-CLEAR-LLM guidelines [39].

## Results

**Fig. 1:**
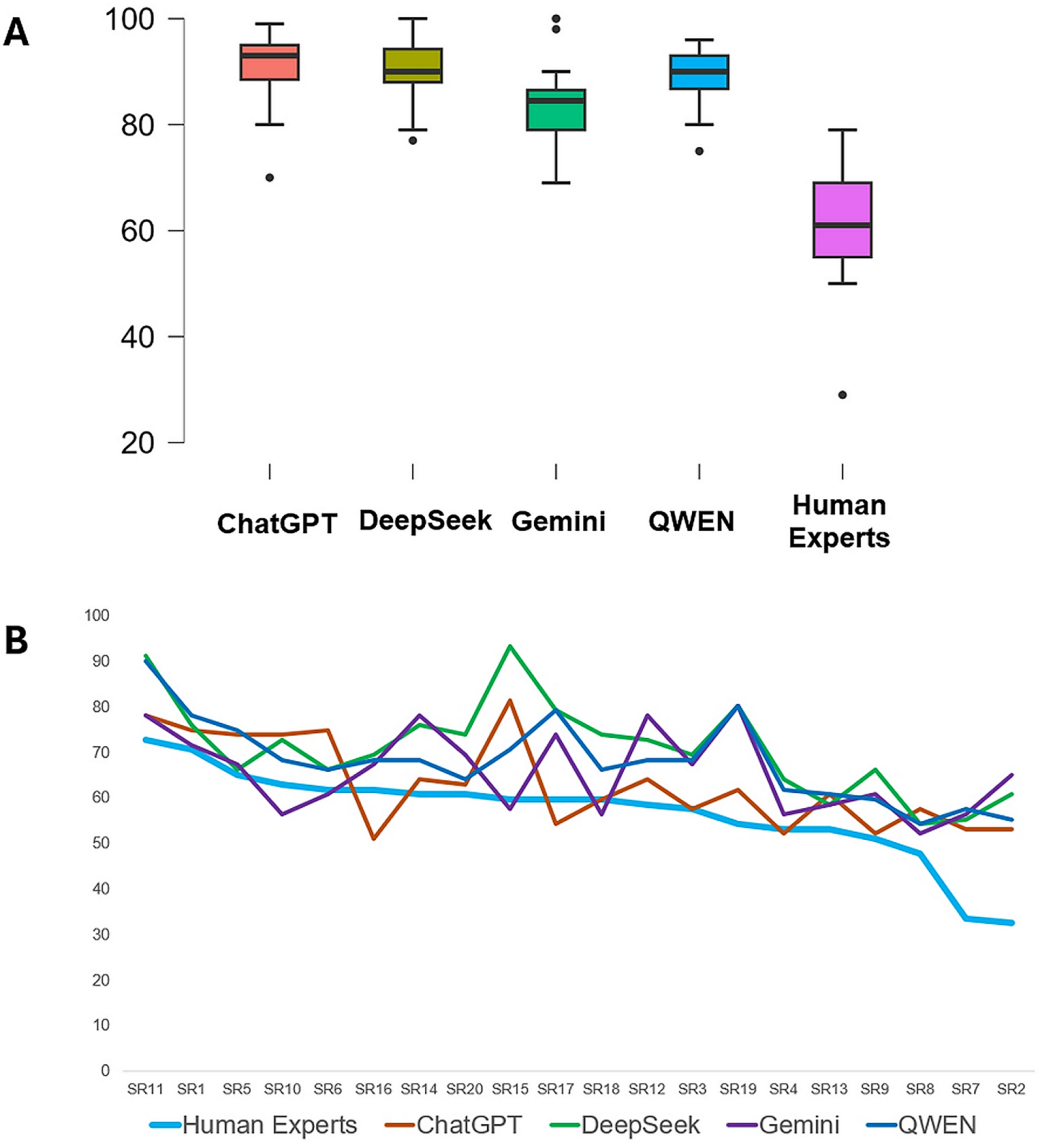
Analysis of the performance of four LLMs for the estimation of adherence to PRISMA 2020, in a sample of SRs, in comparison to human experts. **A**. A box plot for the overall adherence percentages to PRISMA 2020 estimated by the four LLMs and by human experts. **B**. A detailed plot of the adherence to PRISMA 2020, estimated by each of the four LLMs and the human experts, for each one of the SRs included

**Fig. 2:**
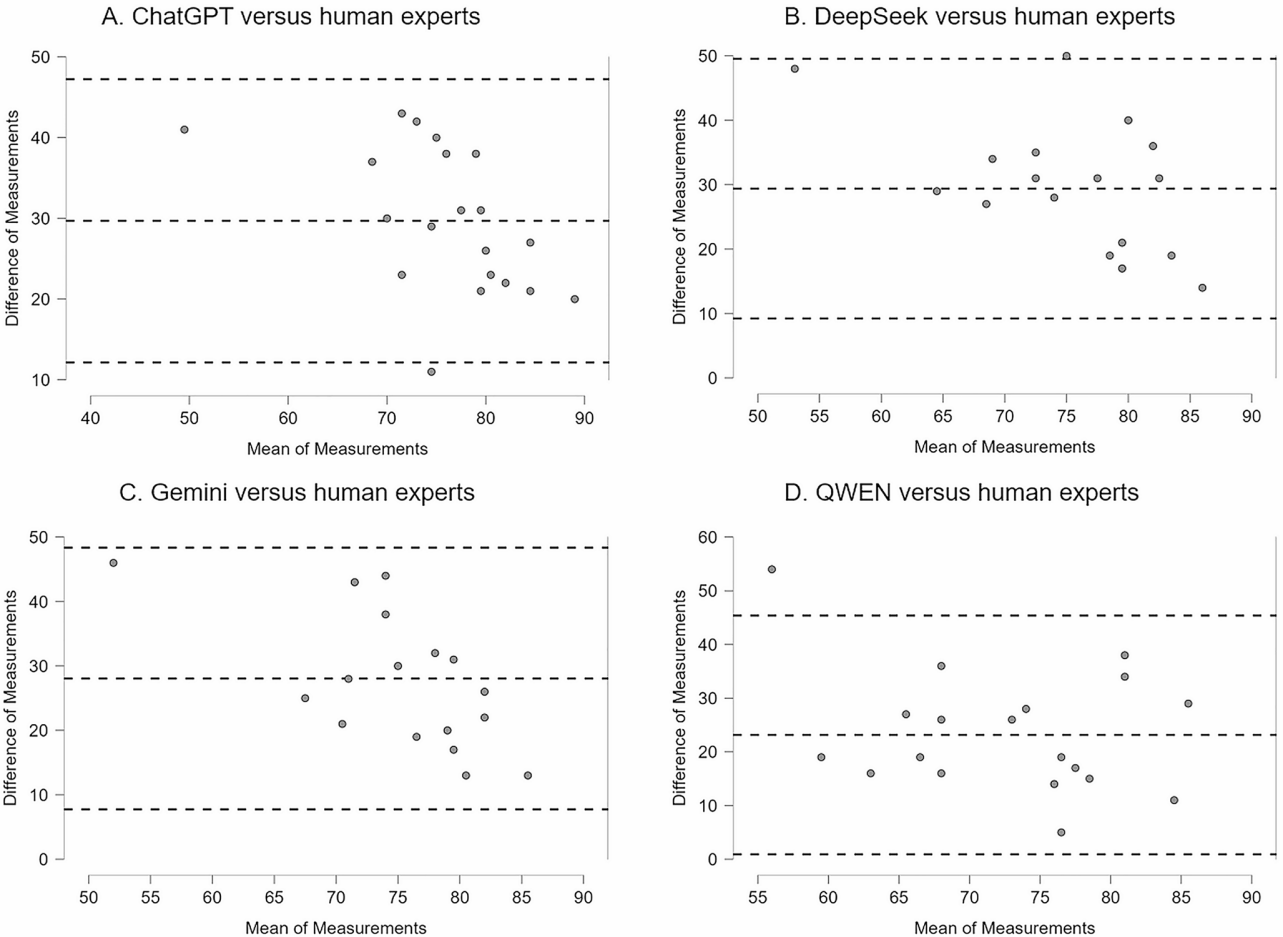
Bland-Altman plots for the concordance of determination of adherence to the PRISMA 2020 statement (overall adherence percentages), for four LLMs in comparison to human experts

For the analysis of the adherence to PRISMA 2020, our analysis identified that there was a higher percentage of adherence in the responses defined by all the four LLMs, in comparison to human subjects: this is shown in box plots (Fig. 1A) and in detailed plots for each one of the included SRs (Fig. 1B). A statistical analysis showed that there were significant differences between each of the LLMs compared to the human experts and that there were no large correlations to the scores defined by human experts (Table 1). An analysis of agreement, using the Bland-Altman plots and tables, showed that the differences between the LLMs and the human experts were large: on average, from 23.1 to 29.7% (Table 1; Fig. 2). The accuracy for each one of the LLMs, for PRISMA 2020, complemented the previously described analyses (Table 3). Overall, these results for PRISMA 2020 indicate a poor performance for all the four tested LLMs.

**Table 1:** Analysis of the performance of four LLMs for the analysis of adherence to PRISMA 2020, in a sample of SRs, in comparison to human experts

Measurement	Adherence (a)	*p* value (b)	Correlation (c)	Bland-Altman (d)
Human Experts	61.2 (11.2)	Taken as reference	Taken as reference	Taken as reference
ChatGPT	90.0 (7.0)	< 0.001	0.60 (0.005)	29.7 (12.2–47.2)
DeepSeek	90.6 (6.8)	< 0.001	0.43 (0.06)	29.4 (9.2–49.6)
Gemini	84.3 (8.5)	< 0.001	0.36 (0.12)	23.2 (0.9–45.4)
Qwen	89.2 (5.6)	< 0.001	0.39 (0.09)	28.1 (7.7–48.4)

(a) Presented as mean (SD) of overall adherence percentages. (b) Results from an ANOVA test, with Tukey´s adjustment, in comparison with human experts. (c) Pearson’s correlation (*p* value in parenthesis), in comparison with human experts. (d) Results from a Bland-Altman analysis: Mean differences (range of difference in parenthesis), in comparison with human experts

An analysis of the adherence to sections of the PRISMA 2020 also found that the differences were large for the multiple sections of items (Table S2) and examples of text showed the differences in the texts of the responses of the LLMs (Table S4).

**Fig. 3:**
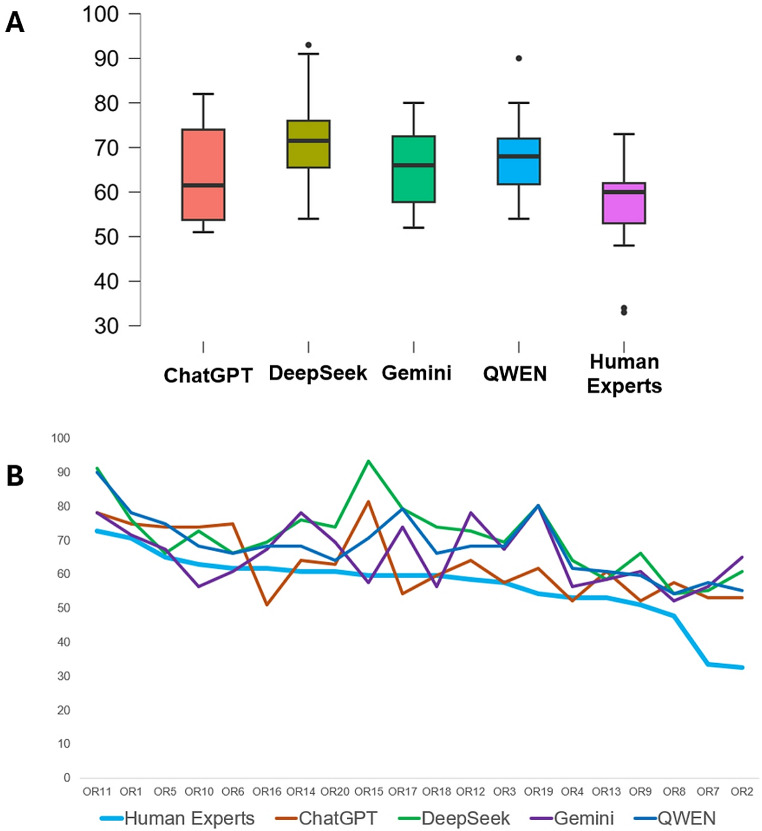
Analysis of the performance of four LLMs for the estimation of adherence to PRIOR, in a sample of overviews of reviews, in comparison to human experts. **A**. A box plot for the overall adherence percentages to PRIOR estimated by the four LLMs and by human experts. **B**. A detailed plot of the adherence to PRISMA 2020, estimated by each of the four LLMs and the human experts, for each one of the overviews of reviews included

**Fig. 4:**
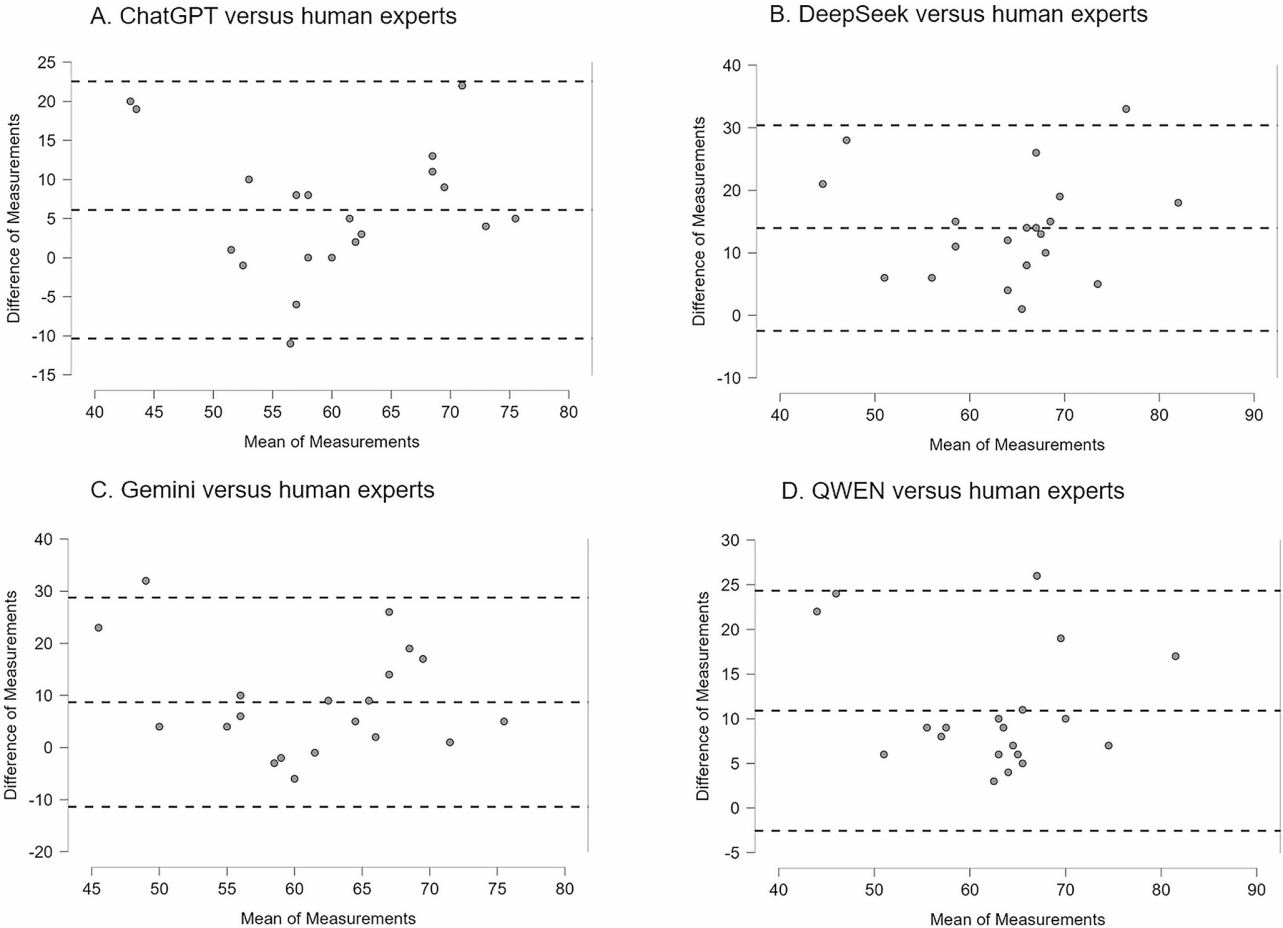
Bland-Altman plots for the concordance of determination of adherence to the PRIOR statement (overall adherence percentages), for four LLMs in comparison to human experts

Regarding the PRIOR statement, our analysis showed that, in comparison to the results from PRISMA 2020, there was better concordance for the adherence defined by the LLMs, in comparison to human experts: this is shown in box plots (Fig. 3A) and in detailed plots for each one of the included overviews of reviews (Fig. 3B). A statistical analysis showed that there were no significant differences between the responses generated by ChatGPT and the scores defined by the human experts and that there was also a significant correlation for ChatGPT and human experts (Table 2). The analysis of agreement, using the Bland-Altman plots and tables, showed that the differences between the ChatGPT and the human experts were smaller: on average, 6.1 (Table 2; Fig. 4). The accuracy for each one of the LLMs, for PRIOR, complemented the previously described analyses (Table 3). Overall, these results for PRIOR indicate a poor performance for three of the four tested LLMs.

**Table 2:** Analysis of the performance of four LLMs for the analysis of adherence to PRIOR, in a sample of overviews of reviews, in comparison to human experts

Measurement	Adherence (a)	*p* value (b)	Correlation (c)	Bland-Altman (d)
Human Experts	57.1 (10.1)	Taken as reference	Taken as reference	Taken as reference
ChatGPT	63.2 (9.9)	0.27	0.65 (0.002)	6.1 (-10.3-22.5)
DeepSeek	71.0 (10.3)	< 0.001	0.66 (0.001)	14.0 (-2.5-30.4)
Gemini	65.8 (8.7)	0.04	0.41 (0.07)	8.7 (-11.4-28.8)
Qwen	68.0 (9.0)	0.005	0.75 (< 0.001)	10.9 (-2.5-24.3)

(a) Presented as mean (SD) of overall adherence percentages. (b) Results from an ANOVA test, with Tukey´s adjustment, in comparison with human experts. (c) Pearson’s correlation (*p* value in parenthesis), in comparison with human experts. (d) Results from a Bland-Altman analysis: Mean differences (range of difference in parenthesis), in comparison with human experts

**Table 3:** Mean accuracy of four LLMs for the automatic identification of adherence to the PRISMA 2020 (upper part) and PRIOR (lower part) statements

PRISMA 2020				
LLM	Gemini	DeepSeek	ChatGPT	QWEN
Mean (SD)	70.4 (9.2)	67.3 (9.0)	61.8 (9.3)	60.5 (9.3)
PRIOR				
LLM	DeepSeek	Gemini	QWEN	ChatGPT
Mean (SD)	74.0 (7.2)	72.2 (7.0)	70.8 (7.0)	67.9 (8.2)

Finally, the analysis of the adherence to sections of the PRIOR found that the differences, between ChatGPT and the human experts, were smaller for the Results and Discussion sections (Table S3) and examples of text also showed the differences in the texts of the responses of the LLMs (Table S5).

## Discussion

This is the first report of the performance of four commonly used LLMs for the automatic identification of adherence to PRISMA 2020 and PRIOR, in a sample of SRs/MAs and overviews of reviews. Among these two statements, PRISMA 2020 [10] is one of the most used and highly cited reporting guidelines in the health sciences, with more than 86,000 citations to date.

In our results, all the four LLMs showed a low performance for the analysis of adherence to PRISMA 2020, in comparison to human experts. In general, the LLMs overestimated the percentage of adherence (in addition to exhibiting low accuracy), and it was more evident for SRs with a low percentage. In contrast, for PRIOR, the LLMs presented lower differences in the estimation of adherence and ChatGPT showed a performance similar to human experts. Our current findings are consistent with the results of a recent scoping review highlighting that general-purpose LLMs are not ready for use in research synthesis [24].

A current major challenge, from the perspective of health sciences research, is the presence of errors, commonly defined as hallucinations or confabulations, in the outputs of LLMs [40, 41]. Transparence about the use of genAI models in health sciences research [42] is a major challenge when there is a lack of available information about the articles used for the training of LLMs [43, 44]. In addition, it is possible that some advanced uses of LLMs, such as the evaluation of adherence to PRISMA 2020, require a better performance of the LLMs in functional linguistic competence [45].

Some recent articles have explored the results from LLMs for related tools. Woelfle et al. explored five versions of three LLMs (including Claude and ChatGPT) for the analysis of PRISMA 2009 (an older version of PRISMA; this study was focused in its current version: PRISMA 2020) in a sample of SRs; they found that the accuracy for the LLMs range from 63 to 70% [13]. Roberts et al. explored ChatGPT 3 for the analysis of the CONSORT-A guidelines and found small differences with scores defined by humans [21]. Other recent studies have focused on LLMs and analysis of risk of bias in SRs/MAs and in primary studies [46–48].

Limitations of the current study include the homogenous nature and relatively small sample size of the included studies [22, 28]. Future studies need the use of more heterogeneous and larger samples of studies and the testing of the potential effect of more complex prompts [49].

Future studies of adherence to other reporting guidelines, including additional LLMs, will be helpful in health sciences research. Additionally, future studies of LLMs designed, or fine-tuned [50], for advanced analyses of epidemiological studies and data [24, 51] are needed. Finally, as the majority of research on LLMs and health sciences research has been carried out in the Global North [52], there is a need for further studies in these topics done in the Global South [43].

## Electronic Supplementary Material

Below is the link to the electronic supplementary material.

Supplementary Material 1

Supplementary Material 2

Supplementary Material 3

## Data Availability

Data is provided within the manuscript or supplementary information files.
