# Supplementary material for: Large Language Models and the Analyses of Adherence to Reporting Guidelines in Systematic Reviews and Overviews of Reviews (PRISMA 2020 and PRIOR)
Source: J Med Syst. 2025 Jun 12;49(1):80. doi: 10.1007/s10916-025-02212-0 (PMC12162794; doi:10.1007/s10916-025-02212-0)
Supplement: Supplementary file 1 — Supplementary Material 1 [file 10916_2025_2212_MOESM1_ESM.docx]

| **LLM** | **Model used** | **Company** | **URL** |
| --- | --- | --- | --- |
| Gemini | 2.0 Flash | Google | gemini.google.com |
| ChatGPT | GPT-4o | OpenAI | chatgpt.com |
| DeepSeek | V3 | DeepSeek | chat.deepseek.com |
| QWEN | 2.5 Max | Alibaba Cloud | chat.qwen.ai |

**Table S1.** Overview of the four freely available LLMs tested in this study.

| **Measurement** | **Adh 1-4** | **Adh 5-15** | **Adh 16-22** | **Adh 23-27** |
| --- | --- | --- | --- | --- |
| **Human Experts** | 57.5 (16.4) | 51.2 (16.8) | 74.1 (15.1) | 65 (9.5) |
| **ChatGPT** | 92.5 (8.5) | 93.4 (6) | 94.3 (6.9) | 82 (13.2) |
| **DeepSeek** | 99.4 (2.8) | 94.7 (4.5) | 87 (9.2) | 84 (13.9) |
| **QWEN** | 95.6 (6.1) | 91.8 (5.3) | 89.8 (9.2) | 81.8 (10.2) |
| **Gemini** | 100 (0) | 89.7 (8.3) | 83.2 (11.9) | 70 (20) |

**Table S2.** Analysis of the performance of four LLMs for the analysis of adherence percentage to PRISMA 2020, in a sample of SRs, for different sections and items (1-4 -Title, Abstract and Introduction; 5-15 -Methods-; 16-22 -Results- and 23-37 -Discussion and Other information), in comparison to human experts. Data is presented as the mean (SD).

| **Measurement** | **Adh 1-4** | **Adh 5-14** | **Adh 15-21** | **Adh 22-27** |
| --- | --- | --- | --- | --- |
| **Human Experts** | 95.6 (7.3) | 41 (11) | 50 (12) | 75 (15.5) |
| **ChatGPT** | 80 (12.4) | 57.8 (11.4) | 56.3 (14.1) | 72.5 (13.3) |
| **DeepSeek** | 96.3 (8.2) | 65.7 (13.2) | 64 (16.8) | 77.9 (10.5) |
| **QWEN** | 88.8 (3.8) | 65 (10.3) | 59 (14.3) | 75.2 (9.6) |
| **Gemini** | 100 (0) | 66 (11.7) | 64.2 (15.6) | 55.4 (12.2) |

**Table S3.** Analysis of the performance of four LLMs for the analysis of adherence percentage to PRIOR, in a sample of overviews of reviews, for different sections and items (1-4 -Title, Abstract and Introduction; 5-14 -Methods-; 15-21 -Results- and 22-27 -Discussion and Other information-), in comparison to human experts. Data is presented as the mean (SD).

| **LLM** | **Response** |
| --- | --- |
| ChatGPT | *“Describes data extraction and risk of bias assessment.”* |
| DeepSeek | *“The data extraction process is described, including independent extraction by four authors and resolution of discrepancies through discussion.”* |
| Gemini | *“Four authors extracted data independently and assessed the quality of each article in pairs. They extracted information about author, country, symptoms, study design, sample size, treatment period, acupuncture protocol style, adverse effects, and significant results. Discrepancies were discussed with another author.”* |
| QWEN | *“Data extraction was performed independently by two researchers, with discrepancies resolved through discussion.”* |

**Table S4.** Examples of the outputs of the four LLMs, for the analysis of the item 9 (Data collection process) of the PRISMA 2020, for a systematic review (SR11) defined as having adherence, for that item 9, by human experts.

| **LLM** | **Response** |
| --- | --- |
| ChatGPT | *“Data extraction procedures are well described.”* |
| DeepSeek | *“The data extraction process is described, including the use of standardized forms and independent extraction by two reviewers.”* |
| Gemini | *“The methods used to collect data from reports are described in the "Data extraction" section. Two reviewers independently extracted data using standardized forms, and discrepancies were resolved through discussion and arbitration.”* |
| QWEN | *“Data collection methods are described, including standardized forms and independent extraction by two reviewers.”* |

**Table S5.** Examples of the outputs of the four LLMs, for the analysis of the item 9a (*Describe the methods used to collect data from report*s) of the PRIOR, for an overview of reviews (OvRe15) defined as having adherence, for that item 9a, by human experts.
