# Supplementary material for: Large Language Models and the Analyses of Adherence to Reporting Guidelines in Systematic Reviews and Overviews of Reviews (PRISMA 2020 and PRIOR)
Source: J Med Syst. 2025 Jun 12;49(1):80. doi: 10.1007/s10916-025-02212-0 (PMC12162794; doi:10.1007/s10916-025-02212-0)
Supplement: Supplementary file 2 — Supplementary Material 2 [file 10916_2025_2212_MOESM2_ESM.docx]

**Supplementary File 1.**

**List of systematic reviews (SRs) and overviews of reviews (OvRes) included**

**PRISMA 2020**

| **SRs** | **Reference** |
| --- | --- |
| SR1 | Al-Moraissi EA, Alradom J, Aladashi O, Goddard G, Christidis N. **Needling therapies in the management of myofascial pain of the masticatory muscles: A network meta-analysis of randomised clinical trials.** J Oral Rehabil. 2020 Jul;47(7):910-922. PMID: 32159870.​ |
| SR2 | Baroncini A, Maffulli N, Eschweiler J, Molsberger F, Klimuch A, Migliorini F. **Acupuncture in chronic aspecific low back pain: a Bayesian network meta-analysis.** J Orthop Surg Res. 2022 Jun 20;17(1):319. PMID: 35725480 |
| SR3 | Bäumler P, Zhang W, Stübinger T, Irnich D. **Acupuncture-related adverse events: systematic review and meta-analyses of prospective clinical studies.** BMJ Open. 2021 Sep 6;11(9):e045961. PMID: 34489268 |
| SR4 | Coyle ME, Stupans I, Abdel-Nour K, Ali H, Kotlyarsky M, Lie P, Tekin S, Thrimawithana T. **Acupuncture versus placebo acupuncture for in vitro fertilisation: a systematic review and meta-analysis.** Acupunct Med. 2021 Feb;39(1):20-29. PMID: 33040570 |
| SR5 | Farag AM, Malacarne A, Pagni SE, Maloney GE. **The effectiveness of acupuncture in the management of persistent regional myofascial head and neck pain: A systematic review and meta-analysis**. Complement Ther Med. 2020 Mar;49:102297. PMID: 32147064 |
| SR6 | Yun JM, Lee SH, Cho JH, Won KW, Heo I. **The effects of acupuncture on occipital neuralgia: a systematic review and meta-analysis**. BMC Complement Med Ther. 2020 Jun 3;20(1):171. PMID: 32493452 |
| SR7 | Lenoir D, van Wijk R, Stocks NP, Thomson O, Ushewokunze S, Coppieters MW. **Acupuncture Versus Sham Acupuncture: A Meta-Analysis on Evidence for Longer-term Effects of Acupuncture in Musculoskeletal Disorders**. Clin J Pain. 2020 Jul;36(7):511-519. PMID: 32028381.​ |
| SR8 | Liu W, Wang CC, Lee KH, Ma X, Kang TL. **Efficacy and Safety of Acupuncture and or Moxibustion for Managing Primary Dysmenorrhea: A Systematic Review and Meta-Analysis.** Clin Nurs Res. 2022 Sep;31(7):1362-1375. PMID: 35499150 |
| SR9 | Llurda-Almuzara L, Labata-Lezaun N, Meca-Rivera T, Navarro-Santana MJ, Cleland JA, Fernández-de-Las-Peñas C, Pérez-Bellmunt A. **Is Dry Needling Effective for the Management of Plantar Heel Pain or Plantar Fasciitis? An Updated Systematic Review and Meta-Analysis.** Pain Med. 2021 Jul 25;22(7):1630-1641. |
| SR10 | Valera-Calero JA, Fernández-de-las-Peñas C, Navarro-Santana MJ, Plaza-Manzano G. **Efficacy of Dry Needling and Acupuncture in Patients with Fibromyalgia: A Systematic Review and Meta-Analysis**. Int J Environ Res Public Health. 2022 Aug 11;19(16):9904. PMID: 36011540. |
| SR11 | Li H, Schlaeger JM, Jang MK, Lin Y, Park C, Liu T, Sun M, Doorenbos AZ. **Acupuncture Improves Multiple Treatment-Related Symptoms in Breast Cancer Survivors: A Systematic Review and Meta-Analysis.** J Altern Complement Med. 2021 Dec;27(12):1084-1097. PMID: 34449251 |
| SR12 | Chang H, Lee H, Kim H, Chung WS**. The Use of Acupuncture in the Management of Patients With Humeral Fractures: A Systematic Review and Meta-analysis**. J Manipulative Physiol Ther. 2021 Feb;44(2):146-153. PMID: 33431276 |
| SR13 | Navarro-Santana MJ, Sanchez-Infante J, Gómez-Chiguano GF, Cummings M, Fernández-de-Las-Peñas C, Plaza-Manzano G. **Effects of manual acupuncture and electroacupuncture for lateral epicondylalgia of musculoskeletal origin: a systematic review and meta-analysis.** Acupunct Med. 2021 Oct;39(5):405-422. PMID: 33334116 |
| SR14 | Han KH, Cho KH, Han C, Cui S, Lin L, Baek HY, Kim J. **The effectiveness and safety of acupuncture treatment on sciatica: A systematic review and meta-analysis.** Complement Ther Med. 2022 Dec;71:102872. PMID: 35985442 |
| SR15 | Jang S, Ko Y, Sasaki Y, Park S, Jo J, Kang NH, Yoo ES, Park NC, Cho SH, Jang H, Jang BH, Hwang DS, Ko SG. **Acupuncture as an adjuvant therapy for management of treatment-related symptoms in breast cancer patients: Systematic review and meta-analysis (PRISMA-compliant).** Medicine (Baltimore). 2020 Dec 11;99(50):e21820. PMID: 33327222 |
| SR16 | Höxtermann MD, Esch HL, Roll S, Brinkhaus B, Icke K. **Safety of acupuncture in oncology: A systematic review and meta-analysis of randomized controlled trials**. Cancer. 2022 May 15;128(10):2001-2016. PMID: 35262912. |
| SR17 | Zhao FY, Fu QQ, Kennedy GA, Conduit R, Zhang WJ, Wu WZ, Zheng Z. **Can acupuncture improve objective sleep indices in patients with primary insomnia? A systematic review and meta-analysis**. Sleep Med. 2021 Apr;80:244-259. PMID: 33610071 |
| SR18 | von Trott P, Oei SL, Ramsenthaler C. **Acupuncture for Breathlessness in Advanced Diseases: A Systematic Review and Meta-analysis**. J Pain Symptom Manage. 2019 Dec;58(6):1021-1039.e1. PMID: 31539602 |
| SR19 | Jo HR, Noh EJ, Oh SH, Choi SK, Sung WS, Choi SJ, Kim DI, Hong SU, Kim EJ. **Comparative effectiveness of different acupuncture therapies for neck pain.** Medicine (Baltimore). 2022 Aug 19;101(33):e29656. PMID: 35984173 |
| SR20 | Jang A, Brown C, Lamoury G, Morgia M, Boyle F, Marr I, Clarke S, Back M, Oh B. **The Effects of Acupuncture on Cancer-Related Fatigue: Updated Systematic Review and Meta-Analysis**. Integr Cancer Ther. 2020 Jan-Dec;19:1534735420949679. PMID: 32996339 |

**PRIOR**

| **OvRes** | **Reference** |
| --- | --- |
| OvRe1 | He W, Li M, Han X, Zhang W. **Acupuncture for Mild Cognitive Impairment and Dementia: An Overview of Systematic Reviews.** Front Aging Neurosci. 2021 May 14;13:647629. PMID: 34054504 |
| OvRe2 | Lee MS, Ernst E. **Acupuncture for surgical conditions: an overview of systematic reviews.** Int J Clin Pract. 2014 Jun;68(6):783-9. PMID: 24447388 |
| OvRe3 | Araya-Quintanilla F, Cuyúl-Vásquez I, Gutiérrez-Espinoza H. **Does acupuncture provide pain relief in patients with osteoarthritis knee? An overview of systematic reviews.** J Bodyw Mov Ther. 2022 Jan;29:117-126. PMID: 35248259 |
| OvRe4 | Zhang XT, Li XY, Zhao C, Hu YY, Lin YY, Chen HQ, Shi ZF, Zhang XY, Shang HC, Tian GH. **An Overview of Systematic Reviews of Randomized Controlled Trials on Acupuncture Treating Migraine**. Pain Res Manag. 2019 Oct 29;2019:5930627. PMID: 31781318 |
| OvRe5 | Yao JP, Chen LP, Xiao XJ, Hou TH, Zhou SY, Xu MM, Wang K, Hou YJ, Zhang L, Li Y. **Effectiveness and safety of acupuncture for treating functional constipation: An overview of systematic reviews.** J Integr Med. 2022 Jan;20(1):13-25. PMID: 34838459 |
| OvRe6 | Lin T, Huang F, Zhao S, Qiu M, Wen J, Liu M. **Acupuncture for diabetic peripheral neuropathy: An overview of systematic reviews.** Complement Ther Clin Pract. 2021 May;43:101375. PMID: 33819833 |
| OvRe7 | Smith CA, Carmady B. **Acupuncture to treat common reproductive health complaints: An overview of the evidence.** Auton Neurosci. 2010 Oct 28;157(1-2):52-6. PMID: 20483671 |
| OvRe8 | Chan MWC, Wu XY, Wu JCY, Wong SYS, Chung VCH. **Safety of Acupuncture: Overview of Systematic Reviews.** Sci Rep. 2017 Jun 13;7(1):3369. PMID: 28611366 |
| OvRe9 | Selva Olid A, Martínez Zapata MJ, Solà I, Stojanovic Z, Uriona Tuma SM, Bonfill Cosp X. **Efficacy and Safety of Needle Acupuncture for Treating Gynecologic and Obstetric Disorders: An Overview**. Med Acupunct. 2013 Dec 1;25(6):386-397. PMID: 24761184 |
| OvRe10 | Qi WC, Fu HJ, Sun RR, Li X, Cai DJ, Wang C, Liang FR. **Effectiveness and safety of acupuncture for angina pectoris: An overview of systematic reviews.** Integr Med Res. 2022 Sep;11(3):100864. PMID: 35535308 |
| OvRe11 | Wang X, Wang Y, Wei S, He B, Cao Y, Zhang N, Li M. **An Overview of Systematic Reviews of Acupuncture for Infertile Women Undergoing in vitro Fertilization and Embryo Transfer**. Front Public Health. 2021 Apr 20;9:651811. PMID: 33959581 |
| OvRe12 | Li J, Li YX, Luo LJ, Ye J, Zhong DL, Xiao QW, Zheng H, Geng CM, Jin RJ, Liang FR. **The effectiveness and safety of acupuncture for knee osteoarthritis: An overview of systematic reviews.** Medicine (Baltimore). 2019 Jul;98(28):e16301. PMID: 31305415 |
| OvRe13 | Huang J, Qin X, Cai X, Huang Y. **Effectiveness of Acupuncture in the Treatment of Parkinson's Disease: An Overview of Systematic Reviews.** Front Neurol. 2020 Aug 25;11:917. PMID: 32973668 |
| OvRe14 | Li YX, Xiao XL, Zhong DL, Luo LJ, Yang H, Zhou J, He MX, Shi LH, Li J, Zheng H, Jin RJ. **Effectiveness and Safety of Acupuncture for Migraine: An Overview of Systematic Reviews.** Pain Res Manag. 2020 Mar 23;2020:3825617. PMID: 32269669 |
| OvRe15 | Lu L, Zhang Y, Ge S, Wen H, Tang X, Zeng JC, Wang L, Zeng Z, Rada G, Ávila C, Vergara C, Chen R, Dong Y, Wei X, Luo W, Wang L, Guyatt G, Tang CZ, Xu NG. **Evidence mapping and overview of systematic reviews of the effects of acupuncture therapies**. BMJ Open. 2022 Jun 6;12(6):e056803. PMID: 35667716 |
| OvRe16 | Xi J, Chen H, Peng ZH, Tang ZX, Song X, Xia YB. **Effects of Acupuncture on the Outcomes of Assisted Reproductive Technology: An Overview of Systematic Reviews**. Evid Based Complement Alternat Med. 2018 Sep 20;2018:7352735. PMID: 30327681 |
| OvRe17 | Tian ZY, Liao X, Gao Y, Liang SB, Zhang CY, Xu DH, Liu JP, Robinson N. **An Overview of Systematic Reviews and Meta- analyses on Acupuncture for Post-acute Stroke Dysphagia.** Geriatrics (Basel). 2019 Dec 8;4(4):68. PMID: 31817993 |
| OvRe18 | Cao L, Li X, Li M, Yao L, Hou L, Zhang W, Wang Y, Niu J, Yang K. **The effectiveness of acupuncture for Parkinson's disease: An overview of systematic reviews**. Complement Ther Med. 2020 May;50:102383. PMID: 32444048 |
| OvRe19 | Chen J, Shergis JL, Guo X, Zhang AL, Wang H, Lu C, Xue CC, Xie C. **Acupuncture Therapies for Individuals with Overweight or Obesity: An Overview of Systematic Reviews.** Diabetes Metab Syndr Obes. 2022 May 30;15:1651-1666. PMID: 35669360 |
| OvRe20 | Chen J, Liu A, Zhou Q, Yu W, Guo T, Jia Y, Yang K, Niu P, Feng H. **Acupuncture for the Treatment of Knee Osteoarthritis: An Overview of Systematic Reviews.** Int J Gen Med. 2021 Nov 19;14:8481-8494. PMID: 34848997 |
