# Supplementary material for: Large Language Models and the Analyses of Adherence to Reporting Guidelines in Systematic Reviews and Overviews of Reviews (PRISMA 2020 and PRIOR)
Source: J Med Syst. 2025 Jun 12;49(1):80. doi: 10.1007/s10916-025-02212-0 (PMC12162794; doi:10.1007/s10916-025-02212-0)
Supplement: Supplementary file 3 — Supplementary Material 3 [file 10916_2025_2212_MOESM3_ESM.docx]

**Supplementary file 2**

**Prompt for PRISMA 2020**

Please assess the adherence to the PRISMA 2020 statement of the attached systematic review. Please present the results as a table, with three columns (items, assessment and comments). Please include all 27 items and its 42 subitems (subitems such as: 10a, 10b, 13a, 13b, 13c, 13d, 13e, 13f, 16a, 16b, 20a, 20b, 20c, 20d, 23a, 23b, 23c, 23d, 24a, 24b, 24c, 25, 26, 27).

Item number Item name

1 Title

2 Abstract

3 Rationale

4 Objectives

5 Eligibility criteria

6 Information sources

7 Search strategy

8 Selection process

9 Data collection process

10a List and define all outcomes for which data were sought.

10b List and define all other variables for which data were sought

11 Study risk of bias assessment

12 Effect measures

13a Describe the processes used to decide which studies were eligible for each synthesis

13b Describe any methods required to prepare the data for presentation or synthesis

13c Describe any methods used to tabulate or visually display results of individual studies and syntheses.

13d Describe any methods used to synthesise results and provide a rationale for the choice(s).

13e Describe any methods used to explore possible causes of heterogeneity among study results

13f Describe any sensitivity analyses conducted to assess robustness of the synthesised results.

14 Reporting bias assessment

15 Certainty assessment

16a Describe the results of the search and selection process

16b Cite studies that might appear to meet the inclusion criteria, but which were excluded,

17 Study characteristics

18 Risk of bias in studies

19 Results of individual studies

20a For each synthesis, briefly summarise the characteristics and risk of bias among contributing studies.

20b Present results of all statistical syntheses conducted.

20c Present results of all investigations of possible causes of heterogeneity among study results.

20d Present results of all sensitivity analyses conducted to assess the robustness of the synthesized results

21 Reporting biases

22 Certainty of evidence

23a Provide a general interpretation of the results in the context of other evidence

23b Discuss any limitations of the evidence included in the review.

23c Discuss any limitations of the review processes used.

23d Discuss implications of the results for practice, policy, and future research.

24a Provide registration information for the review, including register name and registration number,

24b Indicate where the review protocol can be accessed, or state that a protocol was not prepared

24c Describe and explain any amendments to information provided at registration or in the protocol.

25 Support

26 Competing interests

27 Availability of data, code, and other materialsPrompts for AMSTAR 2

**Prompt for PRISMA 2020**

Please assess the adherence to the PRIOR statement of the attached overview of reviews. Please present the results as a table, with three columns (items, assessment and comments). Please include all 27 items and its 46 subitems.

Item number Item name

1 Title

2 Abstract

3 Rationale

4 Objectives

5a Specify the inclusion and exclusion criteria for the overview of reviews.

5b Specify the definition of “systematic review”

6 Information sources

7 Search strategy

8a Describe the methods used to decide whether a systematic review or supplemental primary study (if included) met the inclusion criteria of the overview of reviews.

8b Describe how overlap in the populations, interventions, comparators, and/or outcomes of systematic reviews was identified and managed during study selection.

9a Describe the methods used to collect data from reports.

9b If applicable, describe the methods used to identify and manage primary study overlap at the level of the comparison and outcome during data collection.

9c If applicable, specify the methods used to manage discrepant data across systematic reviews during data collection.

10 Data items

11a Describe the methods used to assess risk of bias or methodological quality of the included systematic reviews.

11b Describe the methods used to collect data on (from the systematic reviews) and/or assess the risk of bias of the primary studies included in the systematic reviews

11c Describe the methods used to assess the risk of bias of supplemental primary studies (if included).

12a Describe the methods used to summarise or synthesise results and provide a rationale for the choice(s).

12b Describe any methods used to explore possible causes of heterogeneity among results.

12c Describe any sensitivity analyses conducted to assess the robustness of the synthesised results.

13 Reporting bias assessment

14 Certainty assessment

15a Describe the results of the search and selection process, including the number of records screened, assessed for eligibility, and included in the overview of reviews, ideally with a flow diagram.

15b Provide a list of studies that might appear to meet the inclusion criteria, but were excluded, with the main reason for exclusion.

16 Characteristics of systematic reviews and supplemental primary studies

17 Primary study overlap

18a Present assessments of risk of bias or methodological quality for each included systematic review.

18b Present assessments (collected from systematic reviews or assessed anew) of the risk of bias of the primary studies included in the systematic reviews.

18c Present assessments of the risk of bias of supplemental primary studies (if included).

19a For all outcomes, summarise the evidence from the systematic reviews and supplemental primary studies (if included). If

19b If meta-analyses were done, present results of all investigations of possible causes of heterogeneity.

19c If meta-analyses were done, present results of all sensitivity analyses conducted to assess the robustness of synthesised results.

20 Reporting biases

21 Certainty of evidence

22a Summarise the main findings, including any discrepancies in findings across the included systematic reviews and supplemental primary studies (if included).

22b Provide a general interpretation of the results in the context of other evidence.

22c Discuss any limitations of the evidence from systematic reviews, their primary studies, and supplemental primary studies (if included) included in the overview of reviews.

22d Discuss implications for practice, policy, and future research (both systematic reviews and primary research).

23a Provide registration information for the overview of reviews, including register name and registration number, or state that the overview of reviews was not registered.

23b Indicate where the overview of reviews protocol can be accessed, or state that a protocol was not prepared.

23c Describe and explain any amendments to information provided at registration or in the protocol. Indicate the stage of the overview of reviews at which amendments were made.

24 Support

25 Competing interests

26a Provide contact information for the corresponding author.

26b Describe the contributions of individual authors and identify the guarantor of the overview of reviews.

27 Availability of data and other materials
